# Editorial: Advanced bioremediation technologies and processes for the treatment of synthetic organic compounds (SOCs), Volume II

**DOI:** 10.3389/fbioe.2023.1224526

**Published:** 2023-06-23

**Authors:** Kunal R. Jain, Chirayu Desai, Eric D. van Hullebusch, Datta Madamwar

**Affiliations:** ^1^ Department of Biosciences, Sardar Patel University, Satellite Campus, Anand, India; ^2^ Department of Environmental Biotechnology, Gujarat Biotechnology University, Gandhinagar, Gujarat, India; ^3^ Université Paris Cité, Institut de Physique du Globe de Paris, CNRS, Paris, France; ^4^ P. D. Patel Institute of Applied Sciences, Charotar University of Science and Technology, Changa, India

**Keywords:** xenobiotic, endocrin disruptors, biocatalysis, bioaugmentation, biostimulation

Synthetic organic compounds (SOCs) became an indispensable element of our modern civilization. The detrimental effect of various SOCs on the ecosphere is now evident, and their daily consumption are at the multi-megaton scale annually, which is also bound to rise with the increase in human population and urbanization (Srivastava et al., 2022). However, dealing with the indiscriminate release of tons of these compounds is one of the most pressing global challenges. The spectrum of use of SOCs (such as dye and dye intermediates, organophosphates and pesticides, polyaromatic hydrocarbons, plastic polymers, per and polyfluoroalkyl substances, flame retardants, organic solvents, pharmaceuticals, endocrine disrupters, and their transformed products) is in turn very diverse (Ojha and Tiwary, 2021; Undas et al., 2023). They are characteristically produced to resist various environmental factors, thus enhancing their shelf-life and preventing their abiotic or biotic degradation (Pande et al., 2020). One of the major limitations in the removal of SOCs from the environment is their molecular structure and chemical diversity, which is much more complex. Their complexity further increases when they are found in mixed streams, which increase the heterogeneity of a polluted ecosystem receiving such contaminated streams. With the available metabolic pool, SOCs are difficult to be metabolized by microorganisms and other living forms. Therefore, the bio-toxicity of SOCs eventually increases, gradually becoming persistent pollutants, which require immediate attention for their removal from the polluted environment (Jain et al., 2021).

Even before focusing on priority pollutants, many methods were experimented for the removal of SOCs from polluted environments. Conventional and advanced bioremediation methods were repeatedly tested, but all were met with limited success because of the enormous volume of the pollutants to be treated along with the heterogeneity of these xenobiotic compounds. The inadequacy of the current technologies is being continuously explored, and next-generation technologies are being developed, leading to hybrid systems integrating physico-chemical and biological approaches for industrial waste treatment. These technologies include novel biocatalytic immobilization methods, phytoreactors, microbial electrochemical technologies, and bioaugmentation as well as biostimulation approaches integrated to form hybrid treatment systems.

Thus, the aim of this Research Topic (as was in the first volume) is to collect the recent advances of technology development in the treatment of synthetic organic compounds. This Research Topic comprises four original research articles that discuss the degradation and remediation process of four distinct synthetic organic compounds.

In a first study, Budeli et al. studied the degradation of endocrine-disrupting compounds (EDCs) by two isolates, *Lysinibacillus* sp. BP1 and *Lysinibacillus* sp. BP2 using laccase mediated biocatalysis process. Using the results of the one-variable-at-a-time (OVAT) approach along with a central composite design (CCD), various parameters were characterized and optimized where the inoculum size and copper concentration were found to have significant effects on laccase activity and estrogen degradation. The study also suggested that beads biocatalysts had a higher potential for recyclability and resistibility as compared to clay-based biocatalysts; however, the net removal difference of estrogen was insignificant among the two methods. The authors concluded that SIGC (silver-impregnated clay granules) biocatalysts might be more useful for large-scale applications because of their antibacterial composite and economic feasibility.

In their study, Lara-Moreno et al. isolated the *Stenotrophomonas indicatrix* CPHE1 strain from soil contaminated with polycyclic aromatic hydrocarbons (PAHs) and sequenced its genome to identify the phenanthrene-degrading genes and compared it with other similar genomes from the existing databases. Through RT-PCR analysis, they found the expression and induction of cysteine dioxygenase (*cysDO*), biphenyl-2,3-diol-1,2-dioxygenase (*bphC*), and aldolase hydratase (*phdG*) in the presence of phenanthrene. To examine the actual phenanthrene degradation, a simulation study was conducted using five soil samples artificially contaminated at a concentration of 50 mg of phenanthrene per kg, and approaches such as biostimulation, bioaugmentation, and the use of 2-hydroxypropyl-β-cyclodextrin (HPBCD) as a bioavailability enhancer were employed. Depending on the soil type, the degradation efficiency varied, but the combination of all three approaches proved most efficient and produced the best possible degradation. The authors concluded that the selection of the best bioremediation strategy depending on the physico-chemical and indigenous microbial properties of polluted soil could be crucial for bioremediation.

In another study, Tang et al. designed a two-compartment biofilm reactor for iturin A production using *Bacillus velezensis* ND through batch, fed-batch, and repeated-batch fermentation. Using polyester fiber with a sphere shape as a carrier material for biofilm production, higher production of iturin A was achieved through batch and fed-batch fermentation (66.7% and 63.3%, respectively) as compared to suspended-cell fermentation. The authors, therefore, concluded that a newly designed biofilm reactor with superior performance could be of great potential for the industrial production of iturin A.

Lin et al. demonstrated the development of layer-by-layer self-assembled silica-based bio-macrocapsules for the removal of toluene. They used micron-sized monodisperse silicon dioxide (SiO_2_) microspheres as sacrificial templates to produce chitosan/polylactic acid (CTS/PLA) bio-microcapsules using the layer-by-layer (LBL) assembly method. Using this composite assembly (i.e., LBL bio-microcapsules (LBMs)), the degradation of toluene and the estimation of toluene-degrading enzyme activity were performed under hostile environmental conditions. The study showed that LBMs had reached 90% removal of toluene in 48 h under harsh environmental conditions, which was found to be significantly higher than free bacteria. The authors, therefore, concluded that encapsulating bacteria with LBL microcapsules could be a feasible method for toluene remediation.

The second volume of the Research Topic highlights new developments in the degradation of different SOCs. The newer technology reported in SOCs degradation was the use of micron-sized monodisperse silica in preparation of layer-by-layer (LBL) microcapsules, the integration of molecular methods with biostimulation and bioaugmentation approaches, and the use of laccase on SICG in aquatic matrices. The Research Topic, while discussing the contemporary research status of bioremediation technologies, has also highlighted the need for further studies on the development of universal technologies for the treatment of synthetic organic compounds.
